# Dichlorido{2-morpholino-*N*-[1-(2-pyri­dyl)ethyl­idene]ethanamine-κ^3^ *N*,*N*′,*N*′′}cadmium

**DOI:** 10.1107/S1600536810043163

**Published:** 2010-10-30

**Authors:** Nurulazimah Ikmal Hisham, Nura Suleiman Gwaram, Hamid Khaledi, Hapipah Mohd Ali

**Affiliations:** a Department of Chemistry, University of Malaya, 50603 Kuala Lumpur, Malaysia

## Abstract

In the title compound, [CdCl_2_(C_13_H_19_N_3_O)], the Cd^II^ ion is five-coordinate, with the *N*,*N*′,*N*′′-tridentate Schiff base ligand 2-morpholino-*N*-[1-(2-pyrid­yl)ethyl­idene]ethanamine and two Cl atoms in a distorted square-pyramidal geometry. In the crystal structure, C—H⋯Cl hydrogen-bonding inter­actions connect the mol­ecules into a three-dimensional network.

## Related literature

For the crystal structures of similar compounds, see: Ikmal Hisham *et al.* (2009 ▶); Cai (2009 ▶).
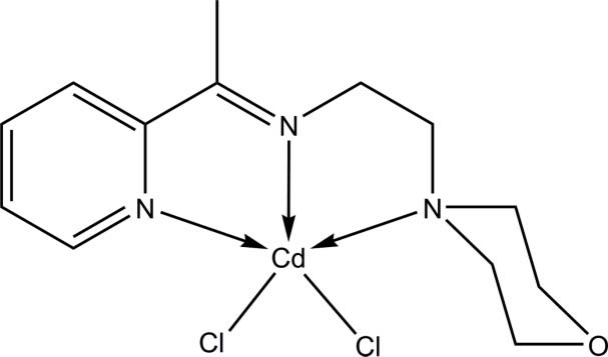


## Experimental

### 

#### Crystal data

[CdCl_2_(C_13_H_19_N_3_O)]
*M* *_r_* = 416.61
Monoclinic, 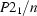

*a* = 9.6357 (12) Å
*b* = 13.9300 (18) Å
*c* = 12.2514 (17) Å
β = 106.776 (2)°
*V* = 1574.5 (4) Å^3^
*Z* = 4
Mo *K*α radiation
μ = 1.73 mm^−1^
*T* = 100 K
0.23 × 0.10 × 0.04 mm

#### Data collection

Bruker APEXII CCD diffractometer
Absorption correction: multi-scan (*SADABS*; Sheldrick, 1996 ▶) *T*_min_ = 0.692, *T*_max_ = 0.934
9407 measured reflections
3437 independent reflections
2646 reflections with *I* > 2σ(*I*)
*R*_int_ = 0.043

#### Refinement

*R*[*F* ^2^ > 2σ(*F* ^2^)] = 0.038
*wR*(*F* ^2^) = 0.117
*S* = 1.08
3437 reflections
182 parameters
H-atom parameters constrained
Δρ_max_ = 0.81 e Å^−3^
Δρ_min_ = −1.16 e Å^−3^

### 

Data collection: *APEX2* (Bruker, 2007 ▶); cell refinement: *SAINT* (Bruker, 2007 ▶); data reduction: *SAINT*; program(s) used to solve structure: *SHELXS97* (Sheldrick, 2008 ▶); program(s) used to refine structure: *SHELXL97* (Sheldrick, 2008 ▶); molecular graphics: *X-SEED* (Barbour, 2001 ▶); software used to prepare material for publication: *SHELXL97* and *publCIF* (Westrip, 2010 ▶).

## Supplementary Material

Crystal structure: contains datablocks I, global. DOI: 10.1107/S1600536810043163/pv2344sup1.cif

Structure factors: contains datablocks I. DOI: 10.1107/S1600536810043163/pv2344Isup2.hkl

Additional supplementary materials:  crystallographic information; 3D view; checkCIF report

## Figures and Tables

**Table 1:** Hydrogen-bond geometry (Å, °)

*D*—H⋯*A*	*D*—H	H⋯*A*	*D*⋯*A*	*D*—H⋯*A*
C4—H4⋯Cl1^i^	0.95	2.69	3.603 (6)	161
C7—H7*C*⋯Cl2^ii^	0.98	2.73	3.607 (6)	149
C8—H8*A*⋯Cl2^iii^	0.99	2.82	3.730 (6)	153
C11—H11*B*⋯Cl1	0.99	2.80	3.654 (6)	144

Symmetry codes: (i) 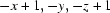; (ii) 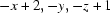; (iii) 
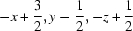
.

**Fig. 1.**
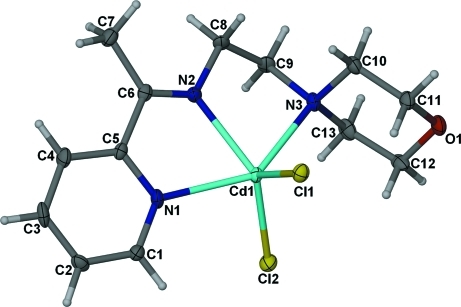
Thermal ellipsoid plot of the title compound at the 50% probability level. Hydrogen atoms are drawn as spheres of arbitrary radius.

## supplementary crystallographic information

### Comment

The title compound has been obtained *via* the complexation of cadmium(II) chloride by the *N,N,N*-tridentate ligand, 2-morpholino-*N*-[1-(2-pyridyl)ethylidene]ethanamine, which had itself been prepared from the condensation of 4-(2-aminoethyl)morpholine and 2-acetylpyridine. The geometry of the complex can be defined as distorted square-pyramidal (τ = 0.18) with one of the chloride ligands in the apical position. Like the similar structures reported in the literature [Ikmal Hisham *et al.*, 2009; Cai, 2009], the morpholine ring in the present complex adopts a chair conformation. In the crystal structure, the molecules are linked together through C—H···Cl interactions into a three dimensional network.

### Experimental

A mixture of 4-(2-aminoethyl)morpholine (0.65 g, 5 mmol) and 2-acetylpyridine (0.61 g, 5 mmol) in ethanol (50 ml) was refluxed for 2 h followed by addition of a solution of cadmium(II) chloride (0.92 g, 5 mmol) in a minimum amount of water. The resulting solution was refluxed for 30 min, then evaporated partially and set aside at room temperature. The crystals of the cadmium(II) complex were obtained after a week.

### Refinement

The hydrogen atoms were placed at calculated positions (C—H 0.95 - 0.99 Å) and were treated as riding on their parent atoms with *U*(H) set to 1.2–1.5 *Ueq*(C).

### Crystal data

**Table:**

[CdCl_2_(C_13_H_19_N_3_O)]	*F*(000) = 832
*M**_r_* = 416.61	*D*_x_ = 1.758 Mg m^−^^3^
Monoclinic, *P*2_1_/*n*	Mo *K*α radiation, λ = 0.71073 Å
Hall symbol: -P 2yn	Cell parameters from 2378 reflections
*a* = 9.6357 (12) Å	θ = 2.3–27.3°
*b* = 13.9300 (18) Å	µ = 1.73 mm^−^^1^
*c* = 12.2514 (17) Å	*T* = 100 K
β = 106.776 (2)°	Lath, colorless
*V* = 1574.5 (4) Å^3^	0.23 × 0.10 × 0.04 mm
*Z* = 4	

### Data collection

**Table:**

Bruker APEXII CCD diffractometer	3437 independent reflections
Radiation source: fine-focus sealed tube	2646 reflections with *I* > 2σ(*I*)
graphite	*R*_int_ = 0.043
φ and ω scans	θ_max_ = 27.0°, θ_min_ = 2.3°
Absorption correction: multi-scan (*SADABS*; Sheldrick, 1996)	*h* = −8→12
*T*_min_ = 0.692, *T*_max_ = 0.934	*k* = −10→17
9407 measured reflections	*l* = −15→15

### Refinement

**Table:**

Refinement on *F*^2^	Primary atom site location: structure-invariant direct methods
Least-squares matrix: full	Secondary atom site location: difference Fourier map
*R*[*F*^2^ > 2σ(*F*^2^)] = 0.038	Hydrogen site location: inferred from neighbouring sites
*wR*(*F*^2^) = 0.117	H-atom parameters constrained
*S* = 1.08	*w* = 1/[σ^2^(*F*_o_^2^) + (0.0488*P*)^2^ + 6.1933*P*] where *P* = (*F*_o_^2^ + 2*F*_c_^2^)/3
3437 reflections	(Δ/σ)_max_ < 0.001
182 parameters	Δρ_max_ = 0.81 e Å^−^^3^
0 restraints	Δρ_min_ = −1.16 e Å^−^^3^

### Special details

**Table:**

Geometry. All e.s.d.'s (except the e.s.d. in the dihedral angle between two l.s. planes) are estimated using the full covariance matrix. The cell e.s.d.'s are taken into account individually in the estimation of e.s.d.'s in distances, angles and torsion angles; correlations between e.s.d.'s in cell parameters are only used when they are defined by crystal symmetry. An approximate (isotropic) treatment of cell e.s.d.'s is used for estimating e.s.d.'s involving l.s. planes.
Refinement. Refinement of *F*^2^ against ALL reflections. The weighted *R*-factor *wR* and goodness of fit *S* are based on *F*^2^, conventional *R*-factors *R* are based on *F*, with *F* set to zero for negative *F*^2^. The threshold expression of *F*^2^ > σ(*F*^2^) is used only for calculating *R*-factors(gt) *etc*. and is not relevant to the choice of reflections for refinement. *R*-factors based on *F*^2^ are statistically about twice as large as those based on *F*, and *R*- factors based on ALL data will be even larger.

### Fractional atomic coordinates and isotropic or equivalent isotropic displacement parameters (Å^2^)

**Table:**

	*x*	*y*	*z*	*U*_iso_*/*U*_eq_	
Cd1	0.77057 (4)	0.09902 (3)	0.33319 (3)	0.01533 (13)	
Cl1	0.52520 (14)	0.14366 (10)	0.21865 (11)	0.0195 (3)	
Cl2	0.96418 (15)	0.21768 (10)	0.36618 (11)	0.0210 (3)	
O1	0.8174 (4)	0.0583 (3)	−0.0120 (3)	0.0235 (9)	
N1	0.7320 (5)	0.1178 (3)	0.5165 (4)	0.0180 (10)	
N2	0.7579 (5)	−0.0471 (3)	0.4170 (4)	0.0173 (10)	
N3	0.8461 (5)	−0.0170 (3)	0.2128 (4)	0.0177 (10)	
C1	0.7366 (6)	0.2002 (4)	0.5727 (4)	0.0194 (11)	
H1	0.7649	0.2567	0.5413	0.023*	
C2	0.7020 (6)	0.2073 (4)	0.6749 (4)	0.0205 (12)	
H2	0.7079	0.2672	0.7131	0.025*	
C3	0.6588 (7)	0.1252 (5)	0.7199 (4)	0.0261 (14)	
H3	0.6321	0.1281	0.7886	0.031*	
C4	0.6552 (6)	0.0378 (4)	0.6629 (5)	0.0214 (12)	
H4	0.6271	−0.0195	0.6927	0.026*	
C5	0.6932 (6)	0.0363 (4)	0.5621 (4)	0.0153 (11)	
C6	0.7049 (6)	−0.0549 (4)	0.4993 (4)	0.0154 (11)	
C7	0.6593 (6)	−0.1485 (4)	0.5389 (5)	0.0225 (12)	
H7A	0.6077	−0.1870	0.4726	0.034*	
H7B	0.5951	−0.1362	0.5866	0.034*	
H7C	0.7452	−0.1836	0.5834	0.034*	
C8	0.7876 (6)	−0.1294 (4)	0.3538 (4)	0.0200 (12)	
H8A	0.6972	−0.1515	0.2978	0.024*	
H8B	0.8274	−0.1830	0.4067	0.024*	
C9	0.8969 (6)	−0.0984 (4)	0.2929 (5)	0.0204 (12)	
H9A	0.9881	−0.0795	0.3504	0.024*	
H9B	0.9188	−0.1538	0.2499	0.024*	
C10	0.7321 (6)	−0.0497 (4)	0.1112 (4)	0.0210 (12)	
H10A	0.6446	−0.0677	0.1334	0.025*	
H10B	0.7662	−0.1074	0.0794	0.025*	
C11	0.6935 (6)	0.0277 (4)	0.0212 (5)	0.0209 (12)	
H11A	0.6187	0.0031	−0.0465	0.025*	
H11B	0.6521	0.0834	0.0511	0.025*	
C12	0.9219 (6)	0.0938 (4)	0.0846 (5)	0.0228 (12)	
H12A	0.8806	0.1489	0.1158	0.027*	
H12B	1.0065	0.1174	0.0619	0.027*	
C13	0.9722 (6)	0.0184 (4)	0.1767 (5)	0.0234 (13)	
H13A	1.0175	−0.0357	0.1473	0.028*	
H13B	1.0456	0.0464	0.2430	0.028*	

### Atomic displacement parameters (Å^2^)

**Table:**

	*U*^11^	*U*^22^	*U*^33^	*U*^12^	*U*^13^	*U*^23^
Cd1	0.0202 (2)	0.0147 (2)	0.01291 (19)	0.00042 (16)	0.00765 (14)	0.00102 (15)
Cl1	0.0196 (6)	0.0200 (7)	0.0194 (6)	0.0030 (5)	0.0065 (5)	0.0031 (5)
Cl2	0.0217 (7)	0.0215 (7)	0.0208 (6)	−0.0038 (5)	0.0076 (5)	0.0009 (5)
O1	0.028 (2)	0.029 (2)	0.0161 (18)	−0.0047 (19)	0.0109 (17)	−0.0021 (17)
N1	0.020 (2)	0.023 (3)	0.011 (2)	0.0020 (19)	0.0050 (18)	0.0010 (18)
N2	0.023 (2)	0.015 (2)	0.014 (2)	0.0009 (19)	0.0050 (18)	0.0000 (18)
N3	0.018 (2)	0.023 (3)	0.013 (2)	0.0012 (19)	0.0057 (18)	−0.0006 (18)
C1	0.025 (3)	0.017 (3)	0.016 (2)	−0.004 (2)	0.005 (2)	0.001 (2)
C2	0.026 (3)	0.016 (3)	0.018 (3)	0.006 (2)	0.004 (2)	−0.004 (2)
C3	0.029 (3)	0.039 (4)	0.010 (2)	0.007 (3)	0.006 (2)	0.002 (2)
C4	0.025 (3)	0.025 (3)	0.017 (3)	0.000 (2)	0.011 (2)	0.003 (2)
C5	0.015 (3)	0.017 (3)	0.013 (2)	0.001 (2)	0.003 (2)	0.004 (2)
C6	0.013 (2)	0.018 (3)	0.015 (2)	0.002 (2)	0.003 (2)	0.003 (2)
C7	0.025 (3)	0.021 (3)	0.021 (3)	−0.002 (2)	0.007 (2)	0.005 (2)
C8	0.032 (3)	0.010 (3)	0.019 (3)	0.006 (2)	0.008 (2)	0.001 (2)
C9	0.029 (3)	0.012 (3)	0.021 (3)	0.009 (2)	0.009 (2)	0.001 (2)
C10	0.026 (3)	0.022 (3)	0.015 (2)	0.000 (2)	0.006 (2)	−0.004 (2)
C11	0.024 (3)	0.024 (3)	0.017 (3)	−0.003 (2)	0.008 (2)	−0.001 (2)
C12	0.026 (3)	0.024 (3)	0.023 (3)	−0.012 (3)	0.014 (2)	−0.008 (2)
C13	0.022 (3)	0.031 (4)	0.021 (3)	0.002 (3)	0.012 (2)	−0.006 (2)

### Geometric parameters (Å, °)

**Table:**

Cd1—N2	2.298 (5)	C4—H4	0.9500
Cd1—N1	2.394 (4)	C5—C6	1.506 (8)
Cd1—N3	2.437 (4)	C6—C7	1.501 (8)
Cd1—Cl2	2.4374 (14)	C7—H7A	0.9800
Cd1—Cl1	2.4557 (14)	C7—H7B	0.9800
O1—C12	1.404 (7)	C7—H7C	0.9800
O1—C11	1.432 (6)	C8—C9	1.519 (8)
N1—C1	1.332 (7)	C8—H8A	0.9900
N1—C5	1.364 (7)	C8—H8B	0.9900
N2—C6	1.261 (7)	C9—H9A	0.9900
N2—C8	1.458 (7)	C9—H9B	0.9900
N3—C10	1.474 (7)	C10—C11	1.510 (8)
N3—C9	1.487 (7)	C10—H10A	0.9900
N3—C13	1.492 (7)	C10—H10B	0.9900
C1—C2	1.390 (7)	C11—H11A	0.9900
C1—H1	0.9500	C11—H11B	0.9900
C2—C3	1.385 (8)	C12—C13	1.515 (8)
C2—H2	0.9500	C12—H12A	0.9900
C3—C4	1.398 (8)	C12—H12B	0.9900
C3—H3	0.9500	C13—H13A	0.9900
C4—C5	1.385 (7)	C13—H13B	0.9900
			
N2—Cd1—N1	68.60 (16)	C6—C7—H7B	109.5
N2—Cd1—N3	75.33 (15)	H7A—C7—H7B	109.5
N1—Cd1—N3	142.63 (15)	C6—C7—H7C	109.5
N2—Cd1—Cl2	131.54 (12)	H7A—C7—H7C	109.5
N1—Cd1—Cl2	95.10 (12)	H7B—C7—H7C	109.5
N3—Cd1—Cl2	101.67 (11)	N2—C8—C9	107.9 (5)
N2—Cd1—Cl1	108.24 (12)	N2—C8—H8A	110.1
N1—Cd1—Cl1	97.11 (11)	C9—C8—H8A	110.1
N3—Cd1—Cl1	103.21 (11)	N2—C8—H8B	110.1
Cl2—Cd1—Cl1	119.18 (5)	C9—C8—H8B	110.1
C12—O1—C11	108.6 (4)	H8A—C8—H8B	108.4
C1—N1—C5	118.5 (4)	N3—C9—C8	113.5 (4)
C1—N1—Cd1	126.0 (4)	N3—C9—H9A	108.9
C5—N1—Cd1	115.4 (3)	C8—C9—H9A	108.9
C6—N2—C8	123.0 (5)	N3—C9—H9B	108.9
C6—N2—Cd1	121.3 (4)	C8—C9—H9B	108.9
C8—N2—Cd1	114.6 (3)	H9A—C9—H9B	107.7
C10—N3—C9	110.2 (4)	N3—C10—C11	111.2 (5)
C10—N3—C13	108.9 (4)	N3—C10—H10A	109.4
C9—N3—C13	107.8 (4)	C11—C10—H10A	109.4
C10—N3—Cd1	115.9 (3)	N3—C10—H10B	109.4
C9—N3—Cd1	101.9 (3)	C11—C10—H10B	109.4
C13—N3—Cd1	111.8 (3)	H10A—C10—H10B	108.0
N1—C1—C2	123.1 (5)	O1—C11—C10	111.7 (5)
N1—C1—H1	118.4	O1—C11—H11A	109.3
C2—C1—H1	118.4	C10—C11—H11A	109.3
C3—C2—C1	118.6 (5)	O1—C11—H11B	109.3
C3—C2—H2	120.7	C10—C11—H11B	109.3
C1—C2—H2	120.7	H11A—C11—H11B	107.9
C2—C3—C4	119.1 (5)	O1—C12—C13	112.4 (5)
C2—C3—H3	120.5	O1—C12—H12A	109.1
C4—C3—H3	120.5	C13—C12—H12A	109.1
C5—C4—C3	118.9 (5)	O1—C12—H12B	109.1
C5—C4—H4	120.5	C13—C12—H12B	109.1
C3—C4—H4	120.5	H12A—C12—H12B	107.9
N1—C5—C4	121.8 (5)	N3—C13—C12	109.7 (5)
N1—C5—C6	115.0 (4)	N3—C13—H13A	109.7
C4—C5—C6	123.1 (5)	C12—C13—H13A	109.7
N2—C6—C7	123.9 (5)	N3—C13—H13B	109.7
N2—C6—C5	116.3 (5)	C12—C13—H13B	109.7
C7—C6—C5	119.8 (4)	H13A—C13—H13B	108.2
C6—C7—H7A	109.5		

### Hydrogen-bond geometry (Å, °)

**Table:**

*D*—H···*A*	*D*—H	H···*A*	*D*···*A*	*D*—H···*A*
C4—H4···Cl1^i^	0.95	2.69	3.603 (6)	161
C7—H7C···Cl2^ii^	0.98	2.73	3.607 (6)	149
C8—H8A···Cl2^iii^	0.99	2.82	3.730 (6)	153
C11—H11B···Cl1	0.99	2.80	3.654 (6)	144

Symmetry codes: (i) −*x*+1, −*y*, −*z*+1; (ii) −*x*+2, −*y*, −*z*+1; (iii) −*x*+3/2, *y*−1/2, −*z*+1/2.
